# Maxillary Bar Overdenture on Immediately Placed Implants: A Clinical Case Report

**DOI:** 10.1155/crid/5908049

**Published:** 2026-09-07

**Authors:** Rubén Jiménez Hernández

**Affiliations:** ^1^ Adjunct Clinical Assistant Professor, Department of Prosthodontics, Faculty of Health Sciences, Catholic University of Murcia (UCAM), Murcia, Spain

**Keywords:** bar-retained overdenture, dental implants, immediate implant placement, implant-supported overdenture, maxilla, primary stability

## Abstract

Bar‐retained maxillary overdentures supported by four implants have shown favorable survival and consistently positive patient‐reported outcomes, particularly when careful prosthetic planning and high primary stability are achieved. The case presented here illustrates the clinical application of this treatment approach as part of a full mouth rehabilitation. A 64‐year‐old male presented with poor esthetics and function due to multiple missing teeth in both arches and nonrestorable maxillary teeth and mandibular molars. Although the mandibular arch could be restored with a combination of fixed crowns and a removable partial denture, the remaining maxillary teeth were all deemed to have a poor prognosis. In shared decision making, the patient opted for removal of these maxillary teeth and replacement with an implant‐supported bar overdenture. Following minimally traumatic extraction and partial grafting of sockets using a xenogeneic bone substitute, four bone‐level implants were placed immediately in the canine–premolar positions (13, 15, 23, and 25) according to a prosthetically driven three‐dimensional plan, achieving insertion torques of 40–45 Ncm. After a 5‐month uneventful osseointegration period and minimally invasive uncovering surgery, straight multiunit abutments and an Ackermann‐type bar (a prosthetic bar design unrelated to the Eckermann implant system) were used to support a bar‐retained maxillary overdenture with bilateral balanced occlusion. At the 4‐ and 7‐month follow‐up visits, peri‐implant tissues were healthy, the implants and bar remained stable, and no biological complications were detected. The patient reported improved comfort, chewing ability, and esthetics. These findings illustrate the short‐term clinical feasibility of this treatment approach in this individual patient.

## 1. Introduction

Advanced partial edentulism of the maxilla associated with severely carious, fractured, or periodontally compromised teeth poses a common clinical challenge [1]. Once the remaining teeth are deemed hopeless, the clinician must present one or more rehabilitation options that balance support, stability, function, and maintenance, ranging from conventional complete dentures to fixed or removable implant‐supported prostheses [1–3].

Immediate implant placement after tooth extraction has been proposed as a strategy to preserve alveolar bone, reduce the number of surgical interventions, and shorten overall treatment time, provided that adequate primary mechanical stability is achieved [4–7]. Factors such as maxillary bone quality, three‐dimensional implant positioning, implant macrogeometry, diameter, and length, as well as the drilling protocol, play a decisive role in obtaining this initial anchorage, which in turn influences implant survival and subsequent prosthetic options [5–7].

In terminal or edentulous maxillae, implant‐supported overdentures retained by a bar provide a favorable balance between support, retention, stability, and hygiene, with predictable outcomes in patient satisfaction and oral health–related quality of life [1–3, 8–12]. Compared with full‐arch fixed prostheses, this approach may be particularly advantageous in patients with limited bone volume and higher maintenance needs, as well as offering acceptable esthetic results [2, 10–12].

Although immediate implant placement and maxillary overdentures have been widely reported, detailed clinical descriptions of the management of compromised maxillae using four immediately placed implants in canine–premolar positions combined with a bar‐retained overdenture remain limited [1, 8, 9, 11]. Moreover, many available reports are based on highly controlled clinical environments or digitally driven workflows, which may limit their applicability in daily practice.

Therefore, this article is aimed at describing the clinical management and short‐term outcomes of maxillary rehabilitation using an Ackermann‐type bar‐retained overdenture supported by four immediately placed implants, emphasizing three‐dimensional planning, symmetrical implant positioning, primary stability, and biomechanical and prosthetic considerations that enhance bar and prosthesis stability. This case report was prepared in accordance with the CARE guidelines for clinical case reporting (File S1).

## 2. Case Report

### 2.1. Patient Information and Chief Complaint

A 64‐year‐old man was referred for a full mouth rehabilitation. He complained of poor esthetics and function due to multiple missing teeth in both arches and broken and mobile maxillary teeth, compromising his ability to chew and smile comfortably. The medical history was noncontributory: The patient reported no systemic diseases, no known drug allergies, and only occasional medication for back pain of unspecified type. The patient expressed a strong preference for a solution that felt as fixed and stable as possible. Although the mandibular arch could be restored with a combination of fixed crowns and a removable partial denture, the remaining maxillary teeth were all deemed to have too poor a prognosis. Based on shared decision making, the patient opted for removal of these maxillary teeth and replacement with an implant‐supported bar overdenture as the most overall suitable and maintainable solution for his situation. The patient provided written informed consent for all clinical procedures and for the publication of anonymized data and images. Clinical images and radiographs of the complete maxillary and mandibular rehabilitation are included for completeness, but the case report in the following is focused on the maxilla. The clinical treatment described in this case report was carried out in a private dental practice.

### 2.2. Clinical and Radiographic Findings

**Figure 1:**
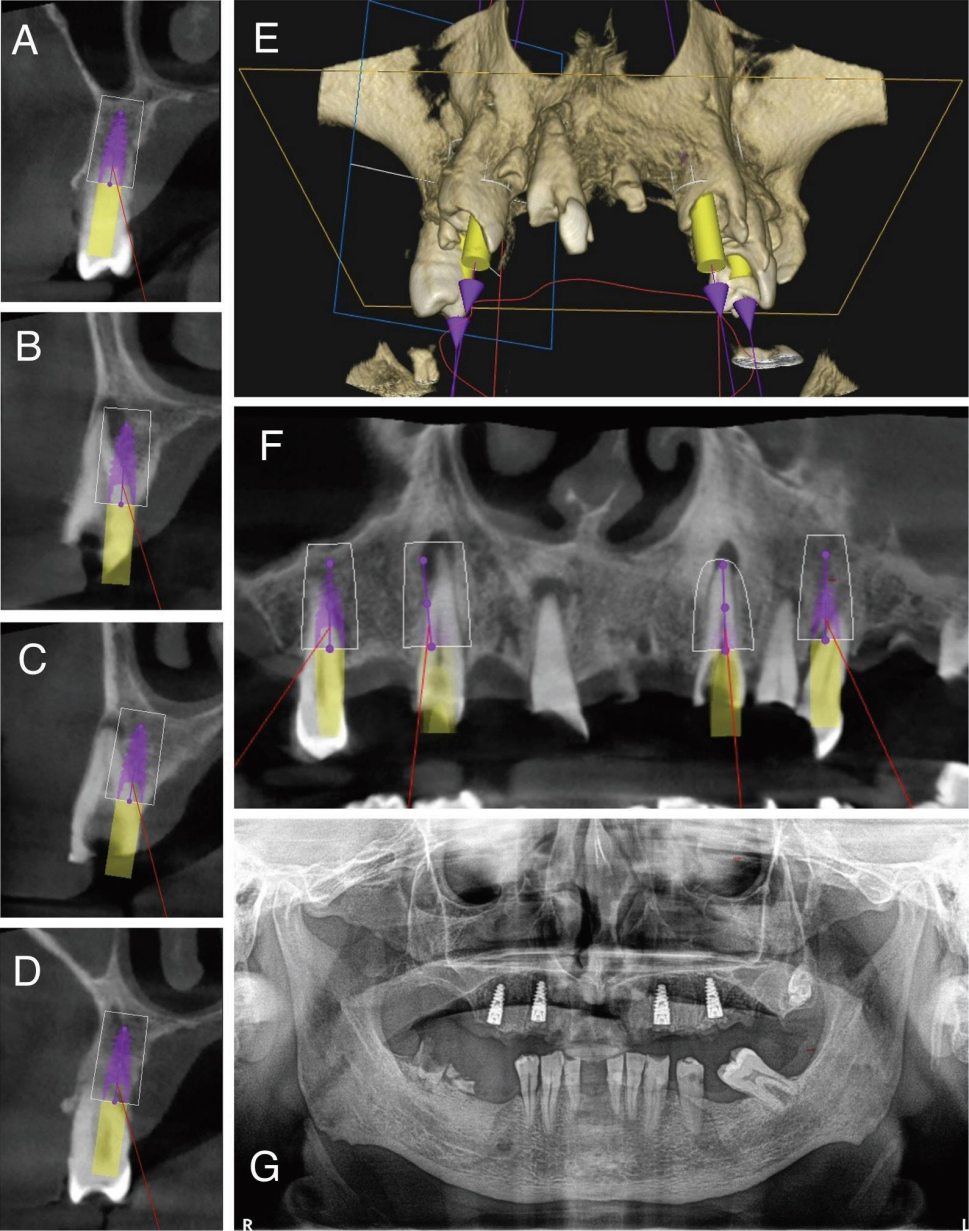
Preoperative planning and immediate surgical outcome. (A–D) Sagittal CBCT slices of the planned canine–premolar implant sites showing apical anchorage. (E) Three‐dimensional reconstruction of four implants in positions 13, 15, 23, and 25. (F) Preoperative panoramic view with virtual implant planning according to the prosthetic design. (G) Postoperative panoramic radiograph confirming symmetrical placement of the four implants.

Intraoral examination revealed a partially edentulous maxilla with several remaining teeth that were severely carious, structurally compromised, or periodontally involved and deemed hopeless. Clinical examination revealed generalized mobility Grade II in all remaining maxillary teeth, with radiographic evidence of periodontal ligament involvement and extensive subgingival caries that compromised most of the coronal structure. Cone‐beam computed tomography (CBCT) confirmed periapical radiolucencies consistent with chronic periapical pathology, without clinical signs of acute infection, along with radiographically evident alveolar bone loss of variable severity across the remaining maxillary teeth. The combination of these findings—significant mobility, periodontal ligament compromise, extensive coronal destruction, and chronic periapical pathology—precluded any predictable prosthetic rehabilitation using these teeth as abutments. Despite this significant bone loss, CBCT also showed sufficient residual bone volume to allow placement of four implants in symmetrical canine–premolar positions (13, 15, 23, and 25) following a prosthetically driven three‐dimensional plan (Figure 1). The mandibular arch was restored with a mixed fixed‐removable prosthetic approach and exhibited a stable occlusal scheme.

### 2.3. Treatment Plan

Following a comprehensive clinical and radiographic evaluation, including intraoral examination and CBCT, the feasibility of immediate implant placement was assessed on a site‐specific basis. Chronic periapical pathology was present at some of the planned extraction sites, without clinical signs of acute infection. CBCT assessment demonstrated sufficient residual apical and palatal bone to permit prosthetically driven three‐dimensional implant positioning and achievement of primary stability. Immediate implant placement was therefore considered feasible, provided that thorough debridement of the extraction sockets was performed following tooth removal, in accordance with current recommendations and published selection criteria for immediate implant placement [4, 13].

The remaining maxillary teeth were considered to have an unfavorable long‐term prognosis as prosthetic abutments. The possibility of preserving the remaining maxillary teeth as abutments for a removable prosthesis was evaluated during treatment planning. However, given the generalized mobility, extensive structural compromise, periodontal involvement, and chronic periapical pathology, their long‐term prognosis as prosthetic abutments was considered unfavorable, and this approach was therefore not considered a predictable treatment option. After discussing treatment options with the patient, including a conventional complete denture and a fixed full‐arch implant‐supported prosthesis, a maxillary implant‐supported overdenture retained by a bar was selected. The plan comprised extraction of all remaining maxillary teeth; immediate placement of four implants in positions 13, 15, 23, and 25; use of a removable interim denture during osseointegration; and fabrication of a definitive bar‐retained overdenture after healing.

The mandibular fixed restorations were splinted to provide a stable and structurally robust prosthetic unit for the bilateral attachments incorporated into the most distal crowns. These attachments provided retention for the removable metal framework prosthesis replacing the posterior missing molar teeth and restoring posterior occlusal function.

### 2.4. Timeline

• October 2024: Extraction of remaining maxillary teeth and immediate placement of four implants in positions 13, 15, 23, and 25.
• March 2025: Second‐stage surgery, placement of multiunit abutments, and definitive impressions.
• April 2025: Delivery of the definitive bar‐retained maxillary overdenture.
• September–December 2025: Clinical and radiographic follow‐up visits, including functional and esthetic assessment.

### 2.5. Surgical Procedure

Under local anesthesia, all remaining maxillary teeth were extracted using a minimally traumatic technique, and granulation tissue was carefully removed. The fresh extraction sockets were partially filled with a xenogeneic bone substitute (Gen‐Os, OsteoBiol, Italy) to compensate for residual defects and support the contours of the buccal plates around the planned implant positions. Four bone‐level implants (Eckermann Triplo, Eckermann, Spain) with a diameter of 4.06 mm and a length of 10 mm were placed immediately in sites 13, 15, 23, and 25 on October 2024 according to the three‐dimensional prosthetic plan, achieving a symmetrical distribution relative to the midline. Site preparation followed a conventional drilling protocol adapted to maxillary bone quality to enhance primary stability; final insertion torques ranged between 40 and 45 Ncm, measured with a calibrated torque wrench. Cover screws were connected to all implants and the sites were closed with tension‐free sutures, leaving the fixtures completely submerged during the osseointegration period. A panoramic radiograph was taken immediately after surgery to verify implant position and seating of the cover screws. No intraoperative or postoperative complications were observed. During the osseointegration period, the patient wore a functional removable interim maxillary denture that remained in occlusion. The tissue‐facing surface of the denture was relined with a permanently soft silicone‐based relining material (Ufi Gel SC, VOCO, Germany) to provide a resilient interface between the rigid acrylic denture base and the healing mucosa over the implant surgical sites, thereby minimizing direct pressure on the operated areas. After an osseointegration period of approximately 5 months, a second, minimally invasive uncovering surgery was performed to expose the implant platforms, remove the cover screws, and allow placement of multiunit abutments prior to initiating the definitive prosthetic phase.

### 2.6. Prosthetic Procedure

**Figure 2:**
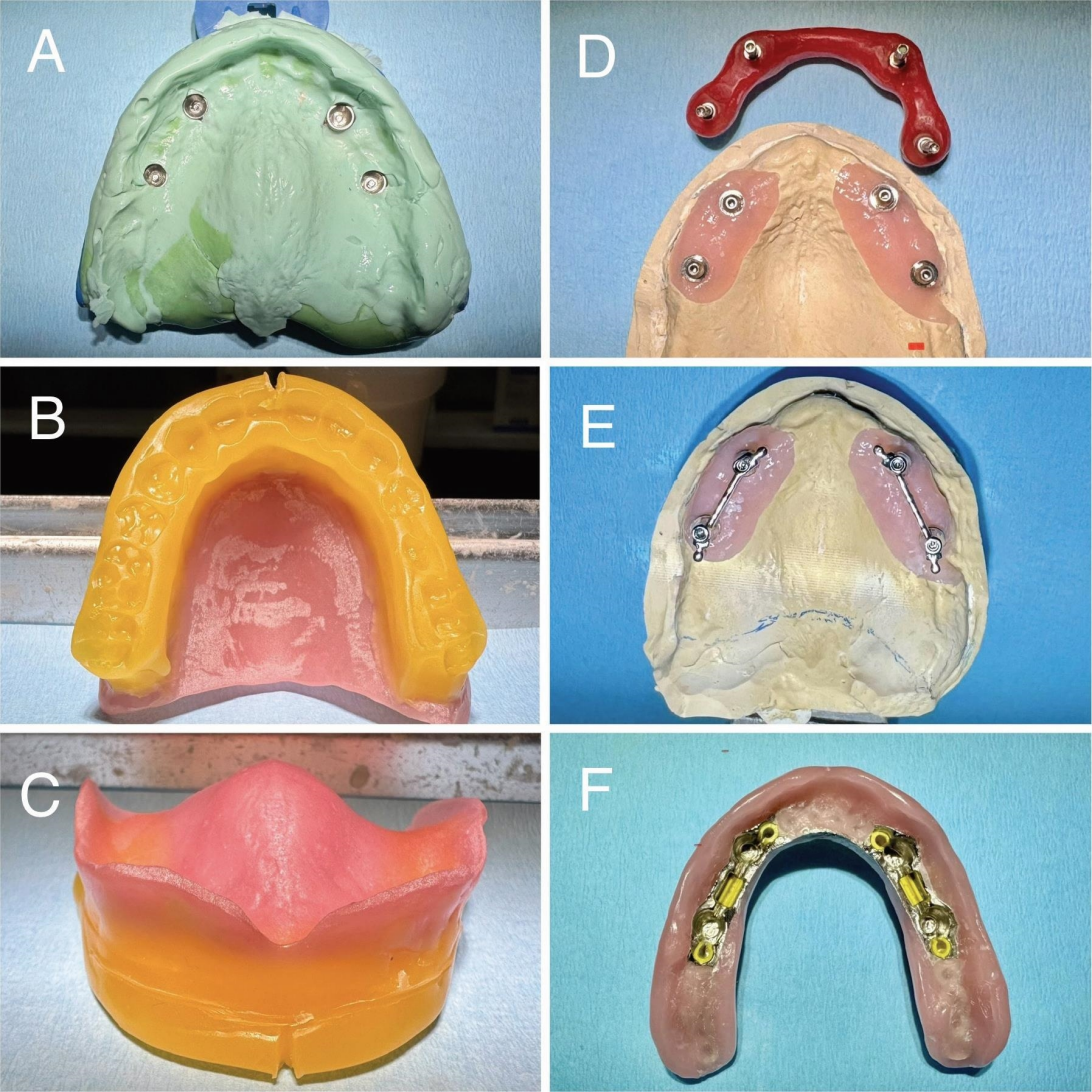
Laboratory steps for fabrication of the bar‐retained maxillary overdenture. (A) Final impression at the multiunit level with open‐tray copings in place, using a polyvinyl siloxane material to capture the healed soft tissues and abutment positions. (B) Definitive maxillary trial denture base and wax rim, adjusted to record maxillomandibular relations and vertical dimension before bar and overdenture fabrication. (C) Frontal view of the processed trial denture, illustrating the planned extension and contour of the future overdenture flange for support and esthetics. (D) Verification jig and resin pattern of the Ackermann‐type bar on the master cast, used to confirm passive fit and to define bar length and cross‐section before casting. (E) Cast framework with bar support areas and clip housings on the master cast, showing the planned distribution of retention along the canine–premolar segments. (F) Intaglio view of the definitive overdenture with metal framework and clip‐type attachments (“riders”) incorporated, ready for connection to the bar.

**Figure 3:**
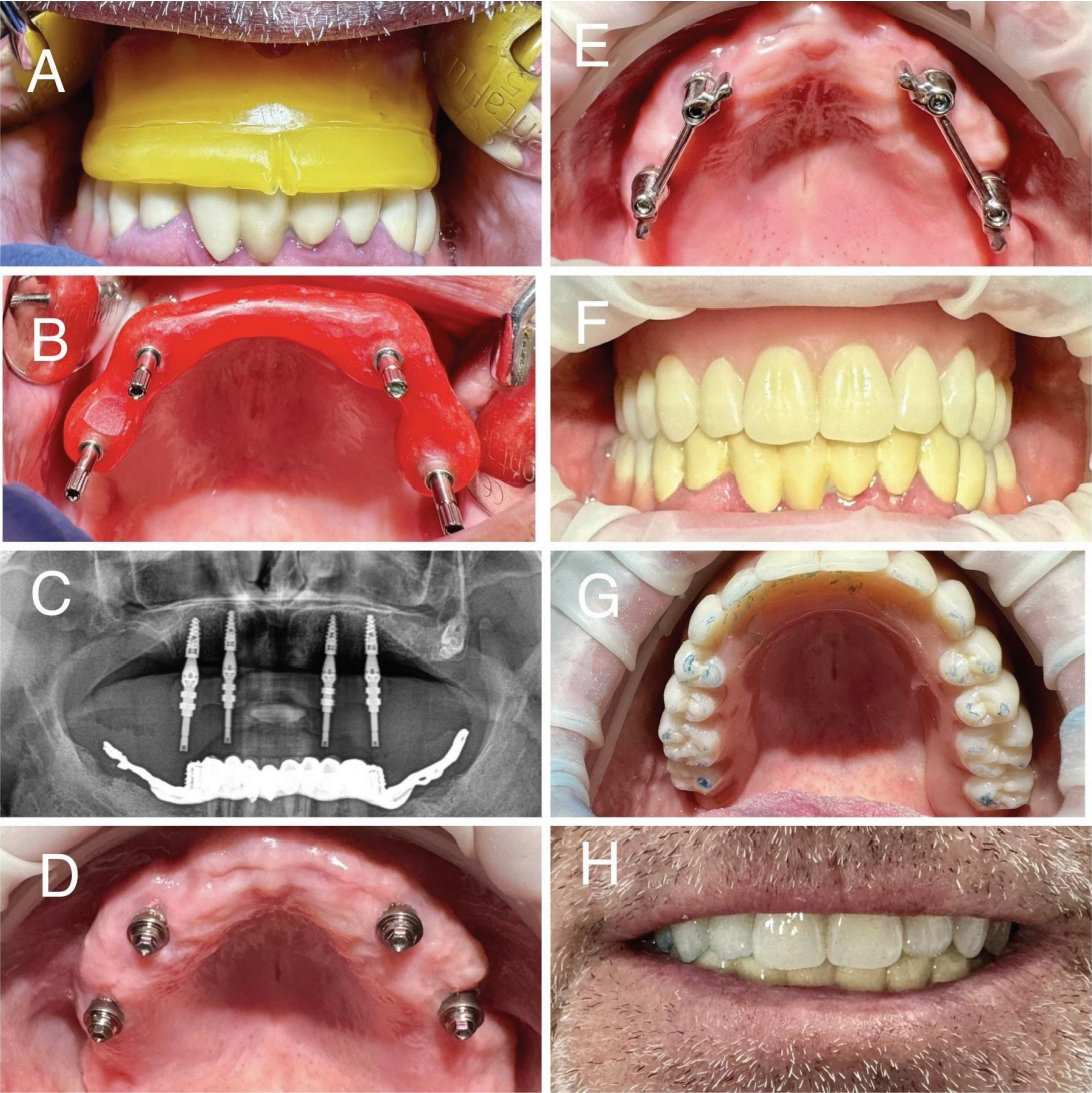
Prosthodontic procedures for the bar‐retained maxillary overdenture. (A) Record base and wax rim adjusted intraorally to register maxillomandibular relations and vertical dimension. (B) Splinted verification jig on multiunit abutments to confirm master‐cast accuracy. (C) Panoramic radiograph showing the verification bar passively seated on the four multiunit abutments. (D) Intraoral view of healed mucosa and emergence profile around the multiunit abutments before bar insertion. (E) Definitive milled Ackermann‐type bar screwed onto the multiunit abutments, with two symmetrical canine–premolar segments. (F) Frontal view of the overdenture during try‐in, verifying esthetics and support. (G) Occlusal view of the definitive overdenture in maximal intercuspidation, showing bilateral balanced contacts. (H) Extraoral view of the patient′s smile with the bar‐retained overdenture in function.

After this uneventful healing period, all four implants showed no detectable clinical mobility at uncovering and no signs or symptoms suggestive of peri‐implant infection or failed osseointegration, and straight multiunit abutments of appropriate height were seated and torqued according to the manufacturer′s recommendations with a torque ratchet (30 Ncm). On March 20, 2025, open‐tray impressions were taken at the abutment level using a polyvinyl siloxane material and a splinted impression technique to ensure accurate transfer of multiunit positions (Figure 2). A DuraLay verification jig, consisting of the impression copings rigidly splinted on the resulting master cast, was subsequently tried intraorally to verify the accuracy of the master cast. A panoramic radiograph was obtained at this stage to confirm complete seating of the verification components on the multiunit abutments (Figure 3). Following satisfactory verification on the master cast, an Ackermann‐type bar in milled cobalt‐chromium alloy was designed with two symmetrical bilateral segments in the canine–premolar regions, each segment incorporating guide tracks at both mesial and distal ends to enhance the path of insertion and overdenture stability; the bar was screw retained on the multiunit abutments, rather than directly on the implant platforms, to facilitate prosthetic access and maintenance. Retention of the overdenture was provided by clip‐type attachments (“riders”) housed in the prosthesis and engaging the bar segments along their length. The definitive overdenture was fabricated on this framework with bilateral balanced occlusion, with even contacts in centric relation and light balancing contacts in lateral excursions. This scheme was selected because of the removable and partially mucosa‐supported nature of the maxillary overdenture, with the aim of maintaining prosthesis stability and minimizing dislodging or rotational movements during eccentric function. The bar‐retained overdenture was delivered on April 23, 2025 after clinical verification of passive fit of the bar, prosthesis seating and stability, adequate function of the internal retentive system, occlusion, esthetics, mucosal support, and lip competence. These findings constituted the clinical baseline assessment at definitive prosthesis delivery. The patient received detailed oral hygiene instructions specific to each prosthetic type: interproximal brushing for the mandibular fixed prosthesis and daily cleaning of the bar, abutments, and intaglio surface of the overdenture using a soft brush and an oral irrigator. A 3‐month maintenance recall schedule was established. The patient demonstrated good adherence to the recommended hygiene protocol, as confirmed at each follow‐up visit.

### 2.7. Follow‐Up and Outcomes

**Figure 4:**
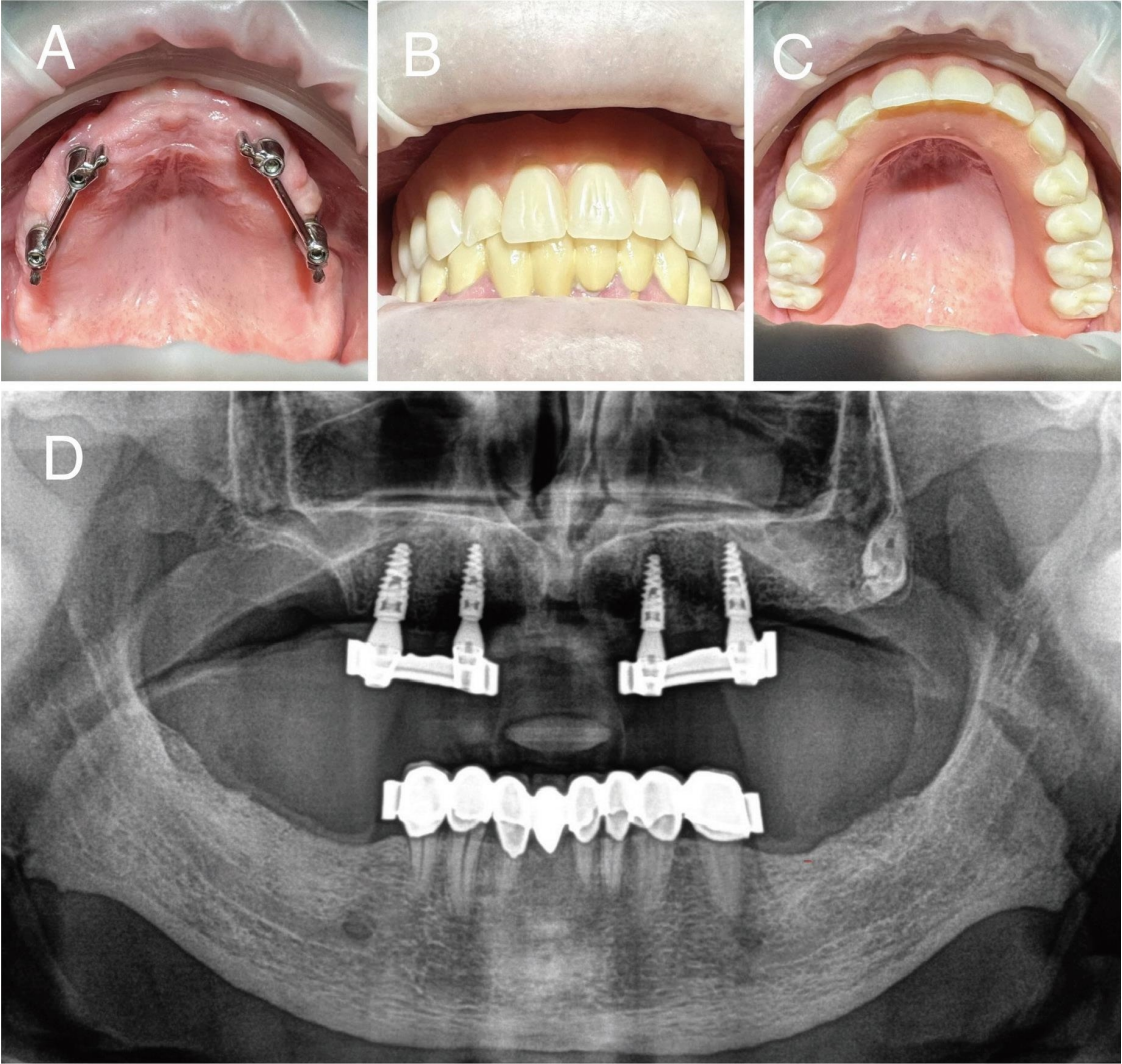
Short‐term clinical and radiographic follow‐up of the bar‐retained maxillary overdenture. (A) Intraoral occlusal view showing healthy peri‐implant soft tissues and the screw‐retained Ackermann‐type bar at follow‐up. (B) Frontal view in maximal intercuspidation illustrating esthetic integration and stable occlusion of the overdenture. (C) Occlusal view of the definitive overdenture in situ, with polished surfaces, bilateral contacts, and adequate extension for support and hygiene. (D) Panoramic radiograph at 7‐month follow‐up showing stable crestal bone around all four implants without radiolucency.

At 4‐ and 7‐month follow‐up visits after delivery of the overdenture (September 3 and December 10 2025), peri‐implant soft tissues were healthy with no evidence of biological complications, and the implants and bar remained stable (Figure 4). The patient reported marked improvement in comfort, chewing ability, and esthetics. During follow‐up, an incisal fracture of the maxillary right lateral denture tooth occurred after the patient reported accidentally biting on an olive pit. The fractured denture tooth was repaired, after which the occlusion was clinically reassessed and adjusted as required. Localized plaque accumulation around the mandibular restorations was managed with professional cleaning and reinforced oral hygiene, including adapted Bass brushing, interproximal cleaning, and use of an oral irrigator [3, 14].

At the 7‐month follow‐up visit, a panoramic radiograph was obtained as an additional radiographic assessment to provide an overall radiographic assessment of the completed full mouth rehabilitation, including both the maxillary implant‐supported prosthesis and the mandibular restorations, rather than in response to any clinical signs or symptoms. No peri‐implant radiolucency or other evident radiographic complications were observed (Figure 4). Implant success was assessed based on the absence of pain, mobility, infection, or prosthetic complications, in accordance with commonly accepted clinical criteria [15]. At the last follow‐up visit, the implants had been in function for approximately 14 months, whereas the bar‐retained overdenture had been in use for about 7 months.

## 3. Discussion

This clinical report describes the rehabilitation of a severely compromised maxilla with a bar‐retained implant overdenture supported by four immediately placed implants in canine–premolar positions. In this patient, this approach provided the most suitable alternative to a fixed full‐arch prosthesis [1, 2, 8, 9, 11, 12]. Although the mandibular arch was restored with a mixed approach—combining fixed metal‐ceramic crowns on the remaining natural anterior teeth with a removable metal framework prosthesis retained by attachments on the distal crowns to replace the missing molar segments—this should not be interpreted as an inconsistency in treatment philosophy. The mandibular anterior teeth presented a favorable prognosis, with adequate bone support and favorable crown‐to‐root ratios, which justified their preservation as fixed abutments. In contrast, the maxillary teeth were deemed nonrestorable due to the severe bone loss, mobility, and structural compromise described above. Moreover, the maxillary bar‐retained overdenture was selected to provide adequate lip support, facilitate peri‐implant hygiene and maintenance, and meet the specific prosthetic requirements of the maxillary arch. This differential, arch‐specific approach, tailored to the varying biological and structural conditions of each jaw, follows the principle of personalized implant‐prosthetic rehabilitation and is consistent with the current evidence on maxillary overdentures. The detailed description provided in this report is intended to enhance understanding of the treatment undertaken in this individual patient.

Evidence from randomized and long‐term studies indicates that maxillary overdentures supported by four implants connected by a bar can achieve survival, peri‐implant tissue health, and patient satisfaction comparable with those supported by six implants, supporting the choice of a four‐implant configuration in this case [8, 9, 11, 12]. However, it should be noted that these randomized clinical trials evaluated maxillary overdentures supported by implants placed in healed ridges rather than immediately after tooth extraction. Therefore, extrapolation of their findings to immediate‐placement protocols is necessarily indirect, as immediate placement presents distinct requirements for achieving adequate primary stability [4, 13].

Although immediate implant placement in the maxilla has traditionally been considered challenging due to lower bone density, anatomical limitations, and the risk of reduced primary stability, recent clinical evidence supports its predictability when strict case selection, careful surgical technique, and adequate insertion torque are achieved [4–7, 13]. Systematic reviews and randomized clinical trials have reported favorable survival rates and peri‐implant tissue outcomes for maxillary overdentures supported by four implants, particularly when combined with prosthetically driven planning and rigid splinting by a bar [1, 5–9]. In this context, the present case describes the application of these principles under routine clinical conditions.

Primary stability was achieved with insertion torques between 40 and 45 Ncm, which fall within or above the commonly recommended threshold of 30–40 Ncm for immediate loading protocols and are considered adequate for predictable osseointegration even in maxillary bone [5–7]. Although immediate loading was not performed in this case, such torque values provide a safety margin for implant–abutment connection stability and may contribute to reduced micromovements during healing [5–7]. The absence of biological complications during the early follow‐up supports the clinical relevance of achieving high primary stability, especially when implants are placed in postextraction sockets in a resorbed anterior maxilla [1, 6–9].

Bilateral balanced occlusion was selected because of the removable and partially mucosa‐supported nature of the maxillary overdenture, with the aim of enhancing prosthesis stability and minimizing dislodging or rotational movements during eccentric function [2]. However, systematic reviews suggest that bilateral balanced occlusion does not necessarily provide superior masticatory performance or quality of life compared with alternative schemes [16]. Accordingly, its use in the present case should be regarded as a clinical choice based on the characteristics of the maxillary removable prosthesis rather than as evidence of superiority over other occlusal schemes.

Although a fully digital workflow could further streamline planning and fabrication, the present case was managed using a conventional analog approach that allowed prosthetically driven implant positioning and bar design [1, 2, 8, 9, 11, 12].

Appropriate patient selection, including adequate residual bone volume, favorable local anatomy, and strong motivation for long‐term oral hygiene and maintenance, remains essential for achieving comparable outcomes.

The main limitations of this report are the single‐case nature and the relatively short prosthetic follow‐up period, which do not allow generalization of the outcomes or evaluation of long‐term marginal bone changes [1, 2, 8, 9, 11, 12]. However, detailed technical reports remain valuable for generating clinically relevant hypotheses and guiding daily clinical practice. Although the prosthetic follow‐up is limited, it corresponds to the early functional phase, during which most biological and mechanical complications associated with maxillary implant placement and prosthetic loading are expected to occur. Therefore, the present observations remain clinically relevant [1, 5–9, 11]. No resonance frequency analysis or standardized radiographic measurements were performed, so quantitative data on implant stability and bone remodeling are lacking and should be addressed in future prospective studies [5–7].

## 4. Conclusions

Within the limitations of this case report, a maxillary bar‐retained overdenture supported by four immediately placed implants in symmetrical canine–premolar positions was associated with stable peri‐implant conditions and satisfactory function and esthetics during short‐term follow‐up [1, 2, 8, 9, 11, 12]. High primary stability at insertion, careful three‐dimensional prosthetic planning, an appropriately designed bar, and a carefully adjusted occlusal scheme may have contributed to the stable short‐term outcome observed in this case [1, 2, 5–9, 16]. This case illustrates the short‐term clinical outcome of this approach undertaken in routine clinical practice in a patient with a compromised maxilla. In the absence to date of prospective studies with longer follow‐up, broader clinical recommendations are available in the previously mentioned, recently published international guidelines for rehabilitation of the edentulous maxilla [3].

## Author Contributions

Rubén Jiménez Hernández: Conceptualization, data curation, formal analysis, investigation, methodology, resources, validation, visualization, writing—original draft, writing—review and editing.

## Funding

No funding was received for this manuscript.

## Disclosure

The author has read and approved the final version of the manuscript. The corresponding author had full access to all of the data in this study and takes complete responsibility for the integrity of the data and the accuracy of the data analysis.

## Ethics Statement

Ethical approval was not required because this manuscript describes a single anonymized clinical case report. This work was conducted in accordance with the principles of the Declaration of Helsinki as revised in 2024. Written informed consent was obtained from the patient for participation in the study and for the publication of anonymized clinical data and accompanying images.

## Conflicts of Interest

The author declares that there is no conflict of interest regarding the publication of this article.

## Supporting information


**Supporting Information** Additional supporting information can be found online in the Supporting Information section. Supporting Information. File S1: CARE Checklist of information to include when writing a case report.

## Data Availability

The author confirms that the data supporting the findings of this study are available within the article and/or its Supporting Information.
